# CoMutDB: the landscape of somatic mutation co-occurrence in cancers

**DOI:** 10.1093/bioinformatics/btac725

**Published:** 2022-11-10

**Authors:** Limin Jiang, Hui Yu, Jijun Tang, Yan Guo

**Affiliations:** Department of Internal Medicine, Comprehensive Cancer Center, University of New Mexico, Albuquerque, NM 87109, USA; Department of Internal Medicine, Comprehensive Cancer Center, University of New Mexico, Albuquerque, NM 87109, USA; Department of Computer Science, University of South Carolina, Columbia, SC, 29201, USA; Department of Internal Medicine, Comprehensive Cancer Center, University of New Mexico, Albuquerque, NM 87109, USA

## Abstract

**Motivation:**

Somatic mutation co-occurrence has been proven to have a profound effect on tumorigenesis. While some studies have been conducted on co-mutations, a centralized resource dedicated to co-mutations in cancer is still lacking.

**Results:**

Using multi-omics data from over 30 000 subjects and 1747 cancer cell lines, we present the Cancer co-mutation database (CoMutDB), the most comprehensive resource devoted to describing cancer co-mutations and their characteristics.

**Availability and implementation:**

The data underlying this article are available in the online database CoMutDB: http://www.innovebioinfo.com/Database/CoMutDB/Home.php.

## 1 Introduction

Somatic mutation is one of the major factors of tumorigenesis (Lawrence *et al.*, 2013). Traditional cancer research studies focus on individual mutations’ functional impact rather than the synergistic mechanisms of multiple concurrent mutations. The phenomenon of multiple mutations occurring in different genes in the same genome is known as co-mutation. Studies have suggested that co-mutation is one of the core determinants of cancers. Co-mutations have been suggested to be associated with pathogenesis, therapeutic vulnerabilities and drug sensitivity in non-small-cell lung cancer (NSCLC) (Skoulidis and Heymach, 2019). Variant co-mutations with KRAS-mutant led to different effects on prognosis and chemotherapy response of NSCLC patients (Arbour *et al.*, 2018). Co-mutations among multiple MAPK pathway genes were reported in a few colorectal cancer patients (Grellety *et al.*, 2016). In a pan-cancer study (Liu *et al.*, 2020), gene pairs’ co-mutation attribute was integrated with gene co-expression topology to prioritize potential prognostic and pharmacogenomics biomarkers. Comprehensive co-mutation analyses showed that certain co-mutation pairs have stronger prognosis prediction power than their individual components (Jiang *et al.*, 2022).

## 2 Materials and methods

CoMutDB processed mutation data from three sources. Firstly, mutation data of 10 147 subjects from The Cancer Genome Atlas (TCGA) were downloaded from the Genomic Data Commons. Four types of survival data (overall, disease-specific, disease-free interval, and progression-free interval) for the TCGA subjects were extracted from TCGA Pan-Cancer Clinical Data Resource (Liu *et al.*, 2018). Secondly, somatic mutation and clinical data of 19 412 cancer subjects were downloaded from International Cancer Genome Consortium (ICGC) data portal. For both TCGA and ICGC, we obtained phenotypical variables for the patients (TCGA: age, sex and race; ICGC: age and sex). Thirdly, somatic mutation and drug sensitivity data of 1747 cell lines and 4690 drug experimental data were obtained from the Cancer Dependency Map (DepMap) project (https://depmap.org/portal/). The numerous subjects or cell lines were grouped by cancer type (TCGA), cohort (ICGC) or tissue site (DepMap), and we kept only datasets with a sample size ≥10.

We organize and store the multi-omics data from three external sources in the background of our database. Given a set of genes, we enumerate the possible gene pairs and set each gene pair as an analysis unit. On one hand, we determine the co-mutation status for the gene pair in each sample (subject or cell line); on the other hand, we obtain the values for phenotypic variable, survival and, if available, drug sensitivity for the same sample. We employ linear, logistic, or multinomial regression to infer associations between the co-mutation and a phenotypic variable that is continuous, binary, or categorical. We conduct Cox proportional hazard regression model to assess the prognostic value of the co-mutation. With DepMap cell-line data, we perform *t*-test to compare drug sensitivity between mutant group and wild-type group. As a result, CoMutDB produces a list of co-mutation gene pairs supplemented by the analysis results from different perspectives. In addition to the main co-mutation analyses, CoMutDB also provides single gene mutation frequency and implements a statistical test of co-mutation frequency via R package biRewire (Gobbi *et al.*, 2014). The formation of co-mutation gene pairs and the foresaid various analyses are computed in real time given the user-supplied gene set.

## 3 Results

**Fig. 1.**
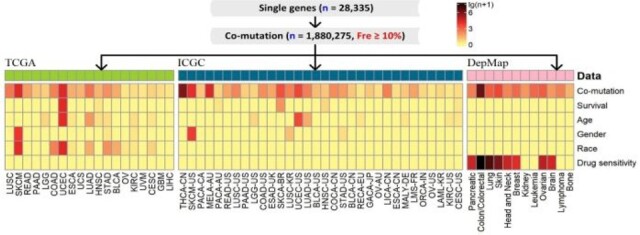
CoMutDB data description. CoMutDB contains somatic co-mutation data mined from three large consortiums (TCGA, ICGC, and DepMap). The heatmap colorcodes the number of pre-computed, downloadable significant co-mutations for each aspect of analyses with respect to a distinct phenotype (survival, age, gender, race, and drug sensitivity), where an eligible co-mutation gene pair must be concurrently mutated in more than 10% samples. In the query service of CoMutDB, the 10% frequency limitation was abolished, and any pair among user-given gene set was treated as a candidate co-mutation. Three top side bars used different colors to differentiate the source data consortiums (TCGA, ICGC, and DepMap). In the heatmap, the color intensivity of each cell is proportional to the number of statistically significant phenotype-associated co-mutation pairs, with color scale illustrated on the top right

The CoMutDB database and its web portal were developed using MySQL, HTML, PHP and Javascript. CoMutDB allows the user to download pre-computed analysis results from our related work (Jiang *et al.*, 2022), where all co-mutation pairs that have more than 10% mutant samples were included. As we explained in the related work (Jiang *et al.*, 2022), co-mutations are defined at two levels: position level and gene level. At position level, any two mutations may be considered as a co-mutation pair as long as it suffices the 10% frequency criterion. At gene level, a pair of genes is considered co-mutated if mutations co-occur in the two genes in more than 10% of samples. Our download portal curates both position-level and gene-level co-mutation datasets. The datasets included 14 440 prognosis-associated, 11 733 sex-associated, 14 896 age-associated, 27 726 race-associated and 76 468 697 drug sensitivity-associated co-mutations (Fig. 1). For real-time query and computation, CoMutDB does not impose the >10% threshold.

Additionally, CoMutDB responds to user queries with real-time computation. Because CoMutDB absorbed multi-omics data from three distinct sources, we implemented a separate query page for each data source, where the user needs to select one or more datasets (cancer types, cohorts, or cell lines) to perform the query. Co-mutation query is enabled at the gene level rather than the position level. The most important input to the query service is a set of genes, from which co-mutation pairs are formed and possible associations with phenotypic variables, survival, and drug sensitivity are examined. CoMutDB can be queried by parameters including cancer type, tissue site, gene name, number of co-mutated genes (up to 4), mutation type (silent, non-silent and all).

The critical step of CoMutDB computation is to divide samples within one dataset into co-mutation mutant and wild-type groups. While a co-mutation typically involves two individual genes, our query service allows a co-mutation entity to comprise up to four genes. While a co-mutation typically refers to ‘concurrent’ mutations of individual genes, we can alternatively assert a co-mutation as long as any component gene is mutated. For a specific co-mutation, a logical operator (‘and’, ‘or’) can be specified to define the relationship between the co-mutated genes. For example, if ‘*TP53* and *KRAS*’ is specified, the samples in the mutant group must exhibit mutations in both *TP53* and *KRAS* genes. If ‘*TP53* or *KRAS*’ is specified, the samples in the mutant group must harbor at least one mutation in either *TP53* or *KRAS*. Upon the division of samples based on co-mutation status, CoMutDB integrates survival data, drug sensitivity, and other clinical characteristics (age, sex, and stage) with the co-mutation status variable, and infers the statistical significance of the association between co-mutation and the various clinical characteristics. The outputs of CoMutDB are in tabular format and can be browsed or downloaded.

The co-occurrence of somatic mutations reflects the concept of epistasis which describes any relationship of non-additive interaction between two or more genes in their combined effect on phenotype. In cancer, random mutations are jointly selected if they confer a fitness advantage over the existing genomic landscape. Co-mutations with positive epistasis result in a stronger fitness than the additive effects of the individual mutations. Consequently, such mutations are more likely to occur together within the same tumor. On the contrary, negative epistasis indicates that co-mutation has weaker fitness than individual effect. Previous studies have shown overwhelming evidence that the acquisition of multiple somatic mutations simultaneously can lead to substantial biological impacts. In many scenarios, the biological impacts can translate into stronger predictions of prognosis and varied drug sensitivity. CoMutDB is the first database dedicated to the curation and description of the landscape of mutation co-occurrence in cancers.

## Funding

This work was supported by the Cancer Center Support [P30CA118100 and R01ES030993-01A1] from the National Cancer Institute, USA.

*Conflict of Interest*: none declared.
